# Add-on Immunoadsorption Shortly-after Optimal Medical Treatment Further Significantly and Persistently Improves Cardiac Function and Symptoms in Recent-Onset Heart Failure—A Single Center Experience

**DOI:** 10.3390/biom8040133

**Published:** 2018-11-02

**Authors:** Karolina Weinmann, Jakob Werner, Wolfgang Koenig, Wolfgang Rottbauer, Daniel Walcher, Mirjam Keßler

**Affiliations:** Department of Internal Medicine II, University Hospital Ulm, 89081 Ulm, Germany; Karolina.weinmann@uniklinik-ulm.de (K.W.); jakob.werner@uni-ulm.de (J.W.); Wolfgang.Koenig@uniklinik-ulm.de (W.K.); Wolfgang.Rottbauer@uniklinik-ulm.de (W.R.); Daniel.Walcher@uniklinik-ulm.de (D.W.)

**Keywords:** immunoadsorption, responsiveness to therapy, cardiomyopathy, heart failure

## Abstract

Background: Immunoadsorption and intravenous immunoglobulin (IVIG) administration may have beneficial effects in patients with dilated cardiomyopathy with end-stage heart failure. We investigated the effect of immunoadsorption with subsequent IVIG administration on cardiac function and symptoms in patients on optimal medical treatment (OMT) for heart failure (HF) with recent-onset cardiomyopathy during long-term follow-up. Methods: Thirty-five patients with recent-onset of HF symptoms received intensive guideline-recommended medical HF therapy for 5.2 months. Subsequently, all patients received a single cycle of immunoadsorption for five days followed by IVIG administration. During the 29-month follow-up period, New York Heart Association (NYHA) functional class, left ventricular ejection fraction (LVEF) and N-terminal pro brain natriuretic peptide (NT-proBNP) were evaluated. Changes in quality of life (QoL) were assessed using the Minnesota Living with HF Questionnaire. Results: Three months after immunoadsorption, NYHA functional class improved from 2.0 to 1.5 (*p* < 0.005) and LVEF significantly increased from 27.0% to 39.0% (*p* < 0.0001). Long-term follow-up of 29 months showed stable NYHA functional class and a further moderate increase in LVEF from 39.0% to 42.0% (*p* < 0.0001) accompanied by a significant improvement in NT-proBNP and QoL scores. Conclusion: Immunoadsorption followed by IVIG administration further enhances LVEF, HF symptoms, QoL and biomarkers in patients with recent-onset HF on OMT.

## 1. Introduction

Dilated cardiomyopathy (DCM) is characterized by impaired ventricular function and chamber enlargement, finally leading to end-stage heart failure (HF). Genetic predisposition, cardiac infection (mostly by cardiotropic viruses) and cardiac inflammatory processes contribute to the pathogenesis of this heterogeneous disease entity [1]. In recent years, numerous antibodies against cardiac antigens have been identified, suggesting an important role of auto-inflammatory processes either in triggering or aggravating the progress of DCM [2,3]. These antibodies most likely arise from a dysregulated host response to infections. Molecular mimicry of viral and host antigens represents one mechanism that may contribute to this autoimmune-mediated myocardial damage [4,5]. Despite the understanding of these underlying pathological mechanisms, an evidence-based, causal treatment option is not yet available [6,7]. Assuming that an infection triggers an autoimmune response against cardiac structures that may initiate recent-onset of DCM and perpetuate progress to end-stage HF, we investigated the effect of early immunoadsorption with subsequent intravenous immunoglobulin (IVIG) administration on cardiac function in patients diagnosed with recent-onset cardiomyopathy on optimal medical HF treatment.

## 2. Methods

### 2.1. Study Population

**Figure 1:**
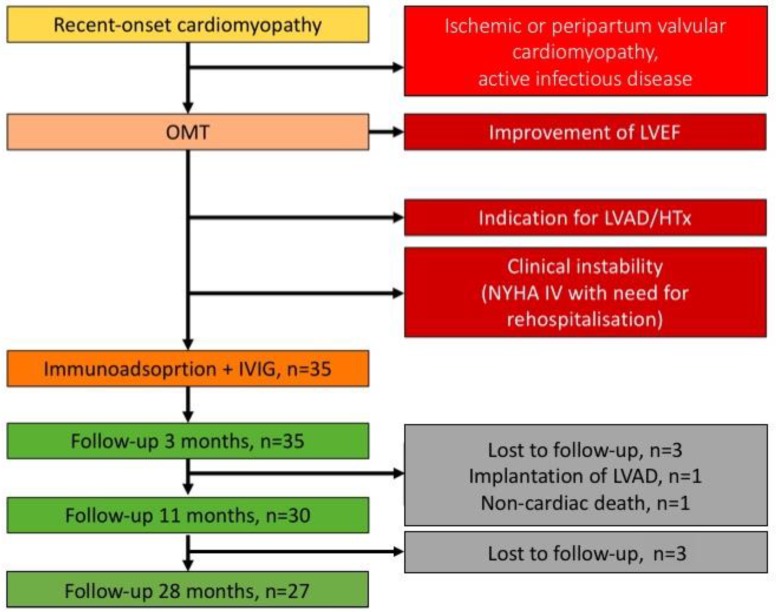
Study design diagram. HTx = heart transplantation.

Thirty-five patients with a recent-onset cardiomyopathy were analyzed. All patients were previously healthy and reported recent-onset of HF symptoms. Inclusion and exclusion criteria are depicted in the diagram (Figure 1). A thorough selection of patients was warranted by an integrated synopsis including history, clinical assessment, laboratory and functional tests [2,8]. These patients received immunoadsorption at our center from October 2011 to April 2016. Patients were followed-up at our institution until December 2016.

### 2.2. Pharmacological Treatment of HF

All patients received optimal medical treatment (OMT) and were treated with guideline-recommended HF therapy consisting of angiotensin converting enzyme (ACE) inhibitors or angiotensin receptor blockers (ARB), beta-adrenergic blocking agents, aldosterone-antagonists and diuretics [9]. Guideline-recommended HF treatment continued throughout the observation period at the maximum tolerated dosage. Prior to immunoadsorption, patients were in a clinically stable condition under OMT.

### 2.3. Diagnostic Approaches at Initial Diagnosis and during Follow-Up Visits

At initial presentation, patients were diagnosed with a recent-onset cardiomyopathy by a careful diagnostic workup. Family history for cardiomyopathy was negative in all cases. The state of health was evaluated by clinical examination, electrocardiogram and laboratory tests in each patient. To exclude other entities of cardiomyopathies, echocardiography, endomyocardial biopsies and selectively, cardiovascular magnetic resonance imaging examinations were performed. An ischemic cardiomyopathy was excluded by cardiac catheterization. Patients with peripartum cardiomyopathy, primary valvular disease and active infectious disease, as well as other significant comorbidities, were excluded (Figure 1). Upon admission to the study, patients were evaluated for New York Heart Association (NYHA) functional class, left ventricular ejection fraction (LVEF), end-diastolic and end-systolic left ventricular diameters (LVDd and LVDs, respectively). After immunoadsorption, follow-up examinations were performed at 3, 10 and 29 months (Figure 1). NYHA functional class, LVEF and LVD (as assessed in four- and two-chamber views by Simpson method) were documented at each visit. In addition, N-terminal pro brain natriuretic peptide (NT-proBNP) levels, as a biomarker of hemodynamic stress, were measured and quality of life (QoL) was assessed before immunoadsorption and during long-term follow-up.

### 2.4. Extracorporal Immunoglobulin Adsorption and Subsequent IVIG Administration

Immunoadsorption therapy was performed, when the patients had achieved a stable condition (NYHA I–IV ambulatory) without improvement in left ventricular function or NYHA class under maximum tolerated heart failure therapy (Figure 1). *Fresenius ART universal*^®^ (Fresenius medical care, Bad Homburg, Germany) immunoadsorber was used. IgG extraction was performed with the commercial Protein-A columns Immunadsorba^®^ (Fresenius Medical Care, Bad Homburg, Germany) with high affinity to IgG1, 2, 4, and low affinity to IgG3, IgA, and IgM [10]. Immunoadsorptive therapy was performed during five consecutive days and IVIG (Privigen^®^, CSL Behring, Marburg, Germany) (0.5 g/kg BW) was administered at day five after immunoadsorption. To determine the success of therapy, a daily serum IgG-level monitoring was performed

### 2.5. Quality of Life (QoL) Assessment Using the Minnesota Living with HF Questionnaire (MLHFQ)

The effects of treatment on QoL were assessed retrospectively using the MLHFQ [11,12]. The MLHFQ is a self-administered questionnaire with 21 items, focusing on functional limitations in the daily life of HF patients. The total score ranges from 0 to 105, with a high score displaying a worse QoL. This score can be subdivided into a physical score of 8 items (range 0–40) and an emotional score of 5 items (range 0–25). The response rate of the QoL by MLHFQ evaluation was 67%.

### 2.6. Evaluation of Responsiveness to Treatment

To evaluate the response to treatment, we introduced two scores, each with a different emphasis regarding the responsiveness to immunoadsorption. First, we used a scoring system, Ohlow et al. had introduced in their study [13]. They defined response to treatment by an improvement of ≥2 parameters of NYHA, LVEF, LVDd, MLHFQ QoL score, NT-proBNP levels and tolerated workload during stress testing. In addition, to focus on maintained changes of cardiac function and myocardial contractility, we assessed the proportion of patients with LVEF improvement of more than 20% of the pre-immunoadsorption value in at least two follow-up examinations.

### 2.7. Statistical Analysis

Statistical analyses were performed using GraphPad Prism 6 Statistics^®^ (GraphPad Software, Inc., La Jolla, CA 92037, USA). Results are presented as median and interquartile range (IQR) because of the non-parametric distribution of data and small sample size, thus avoiding an overestimation of outliners.

Clinical outcomes were analyzed using non-parametric repeated measures analysis. For testing the time course of NYHA functional class, LVEF, LVD and NT-proBNP, Kruskal-Wallis tests were performed. The Wilcoxon matched-pairs test was performed in order to detect specific differences at different time-points during follow-up. A *p*-value < 0.05 was considered statistically significant.

### 2.8. Ethics

The investigation conforms to the principles outlined in the Declaration of Helsinki; written informed consent was obtained from each patient and the protocol was approved by the local hospital’s Ethics Committee.

## 3. Results

### 3.1. Baseline Characteristics of the Study Population

Thirty-five previously healthy patients were identified with recent-onset cardiomyopathy. Inclusion and exclusion criteria are depicted in the diagram (Figure 1). All patients had a negative family history of DCM, valvular heart disease or ischemic cardiomyopathy. Median age at initial diagnosis was 47.1 years (IQR: 35.1 to 53.8). Troponin T levels at initial diagnosis were only mildly elevated (22 ng/L; IQR: 13–34 ng/L), whereas NT-proBNP levels were remarkably increased 5720 ng/L (IQR: 1850–7294 ng/L). Endomyocardial biopsy (EMB) was performed in 27 patients, in which cardiotropic viruses were detectable in 13 patients. A total of 18 patients had received cardiac magnetic resonance imaging (MRI) that fulfilled the diagnostic criteria of myocardial inflammation in 72% of cases. Altogether, 31 patients had received either EMB or cardiac MRI and in 21 (67.7%) patients, myocardial infection or inflammation were detectable (Table 1).

At baseline, all patients presented HF symptoms with a median NYHA functional class of 3.0. Patients showed a median LVEF of 27.0% by echocardiography. LVDs and LVDd demonstrated a dilation of the left ventricle in the majority of patients (Table 2).

Eight patients did not attend all follow-up visits, six patients were lost to follow-up, one patient was excluded because of receiving a left ventricular assist device, and one patient died from non-cardiac reasons. A careful evaluation of these eight patients showed that three improved to a mildly impaired LVEF, three showed an improvement to a moderately impaired LVEF and two persistently had a severe impairment of LVEF at time of loss to follow-up.

### 3.2. Optimal Medical HF Treatment Improved NYHA Functional Class without Affecting LVEF

**Figure 2:**
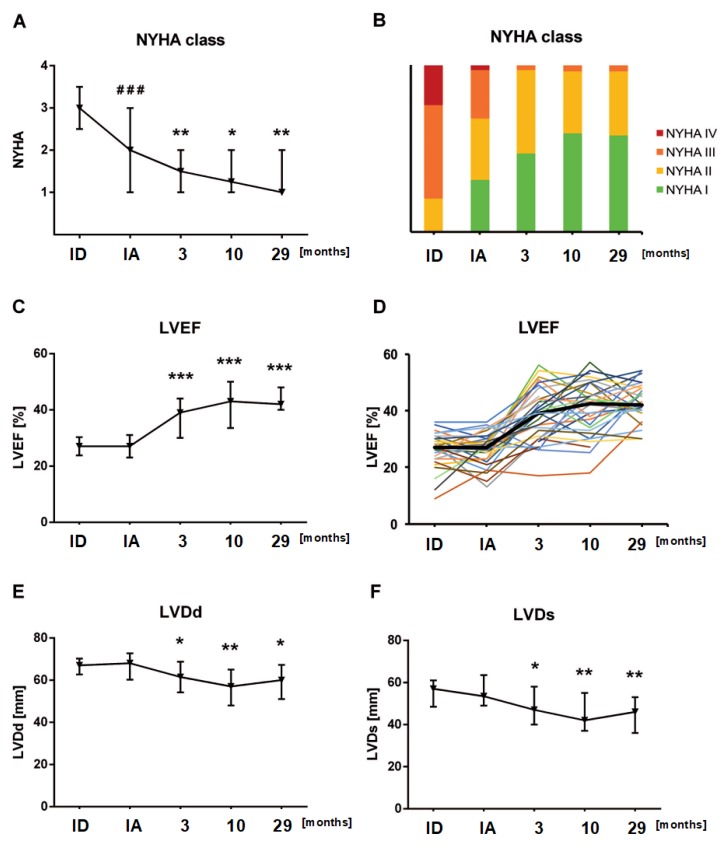
Change of clinical outcome and cardiac performace parameters. Time course of (**A**,**B**) NYHA functional class, (**C**,**D**) left ventricular ejection fraction (LVEF), (**E**) LVDd and (**F**) LVDs are analyzed. Hashes represent significance between initial diagnosis (ID) and immunoadsorption (IA). Asterisks represent significance level between IA and follow-up visits (FU). (### *p* < 0.0001; * *p* < 0.05, ** *p* < 0.005, *** *p* < 0.0001).

Guideline-recommended OMT was started after initial HF diagnosis. The respective equivalent doses at initiation are listed in Table 3. Pharmacological HF treatment was titrated to the maximum tolerated dosage for each individual and was maintained for a median of 5.2 months (IQR: 3.3 to 6.5) before immunoadsorption was initiated. During this period, heart rate decreased significantly from a median of 88.5 (IQR: 75.0 to 99.8) beats per minute (bpm) at initial diagnosis, to a median of 70 (IQR: 62.0 to 74.5; *p* < 0.0001) bpm. NYHA functional class decreased from 3.0 to a steady state of 2.0 (*p* < 0.001). By contrast, median LVEF remained unchanged at 27.0% (*p* = 0.80, Figure 2A,B, Table 2) and LVD decreased moderately (LVDd 67.0 mm to 65.5 mm, *p* = 0.31, LVDs 57.0 mm to 51.0 mm, *p* = 0.50, Table 2). NT-proBNP levels decreased significantly from 5720 ng/L at baseline to 650 ng/L (*p* < 0.0001) during the period of 5.2 months on OMT.

### 3.3. Add-On Immunoadsorption with Subsequent IVIG Administration Improved NYHA Functional Class, LVEF and NT-proBNP during Short-Term and Long-Term Follow-Up

**Figure 3:**
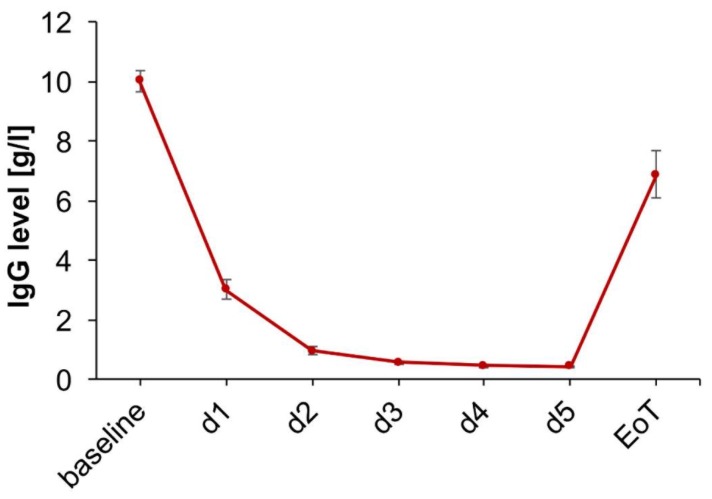
IgG levels during IA therapy. Baseline = before initiation of IA, EoT = end of treatment with intravenous immunoglobulin (IVIG).

Following optimal medical HF therapy, patients were treated with a single cycle of 5-day immunoadsorption (IA) and subsequent IVIG. A daily monitoring of the serum IgG-level demonstrated treatment success. The maximum serum IgG-level reduction was 95.8% (IQR: 95.4 to 96.7%) at the last day of immunoadsorption (Figure 3). Adverse events during the immunoadsorption procedure and IVIG administration were observed in four patients. Two patients had a mild thrombocytopenia without any signs of bleeding and one patient presented with symptomatic hypotension, necessitating fluid therapy during IA. After IVIG administration, one patient showed an allergic reaction with shivering and paleness.

At the first follow-up visit at 3.1 (IQR: 1.8 to 4.7) months after immunoadsorption and IVIG (n = 35 patients), a significant further improvement in NYHA functional class from 2.0 to 1.5 (IQR: 1.0 to 2.0) (vs. at initiation of immunoadsorption, *p* < 0.005) was noted (Figure 2C,D). In addition, a significant increase in LVEF from 27.0% to 39.0% was observed (IQR: 30.5 to 44.0%) (vs. at initiation of immunoadsorption, *p* < 0.0001) (Figure 2A,B), accompanied by a reduction of the dilated left ventricular diameters (LVDd from 65.5 mm (IQR 61.0–68.5) to 61.5 mm (IQR: 54.8–68.3) (vs. at initiation of immunoadsorption, *p* < 0.05) and LVDs from 51.0 mm (IQR 51.0 to 51.0) to 47.0 mm (IQR: 40.5 to 57.0 mm) (vs. at initiation of immunoadsorption, *p* < 0.005)) (Figure 2E,F).

At the second follow-up after 10.2 (IQR: 6.6 to 15.3) months after immunoadsorption (n = 30 patients), NYHA functional class decreased significantly to 1.3 (IQR: 1.0 to 2.0) (vs. at initiation of immunoadsorption, *p* < 0.05), and LVEF further significantly improved to 43.0% (IQR: 34.0 to 50.0%) (vs. at initiation of immunoadsorption, *p* < 0.0001). Likewise, dilated left ventricular diameters decreased (LVDd from 65.5 mm (IQR 61.0–68.5) to 57.0 mm (IQR: 48.5–64.5) (vs. at initiation of immunoadsorption, *p* < 0.005) and LVDs from 51.0 mm (IQR 51.0 to 51.0) to 42.0 mm (IQR: 37.5 to 54.5 mm) (vs. at initiation of immunoadsorption, *p* < 0.005)) (Figure 2E,F).

**Figure 4:**
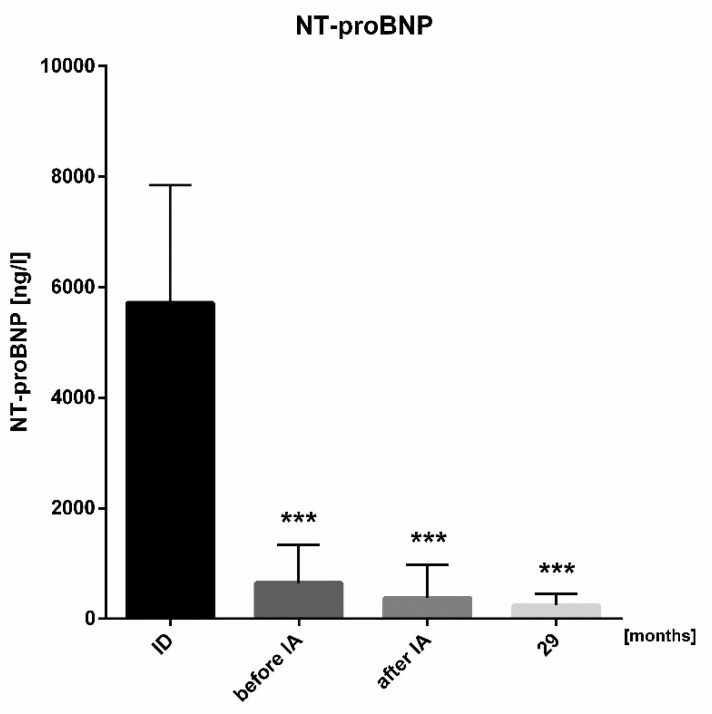
Dynamics of NT-proBNP. NT-proBNP was analyzed at initial diagnosis (ID), on admission for immunoadsorption (IA) on optimal heart failure treatment (before IA), before discharge from hospital (after IA) and at long-term follow-up (n = 24, *** *p* < 0.0001)).

Evaluating long-term effects of immunoadsorption, patients were observed for a median follow-up period of 29.3 (IQR: 14.2 to 41.6) months (n = 27 patients). During the 29-month follow-up period, NYHA functional class, LVEF and LVD remained stable (Figure 2A–F). Throughout long-term follow-up, NT-proBNP levels continuously and significantly decreased (650 ng/L (IQR: 356–1330 ng/L) from initiation of immunoadsorption to 242 ng/L (IQR: 113–452 ng/L), *p* < 0.0001) (Figure 4).

### 3.4. High Response Rate to Immunoadsorption Treatment Regarding Cardiac Function and Clinical Parameters

Using the clinical and echocardiographic criteria of responsiveness to treatment by Ohlow et al., our cohort shows a responsiveness of 100% to IA and IVIG.

To demonstrate a maintained echocardiographic response of left ventricular (LV) contractility, we analyzed a LVEF improvement of ≥20% of the pre-immunoadsorption value at ≥2 follow-up examinations. Thus, 90% of the patients have a maintained LV contractility response.

### 3.5. Significant Improvement in QoL during Long-Term Follow-Up

**Figure 5:**
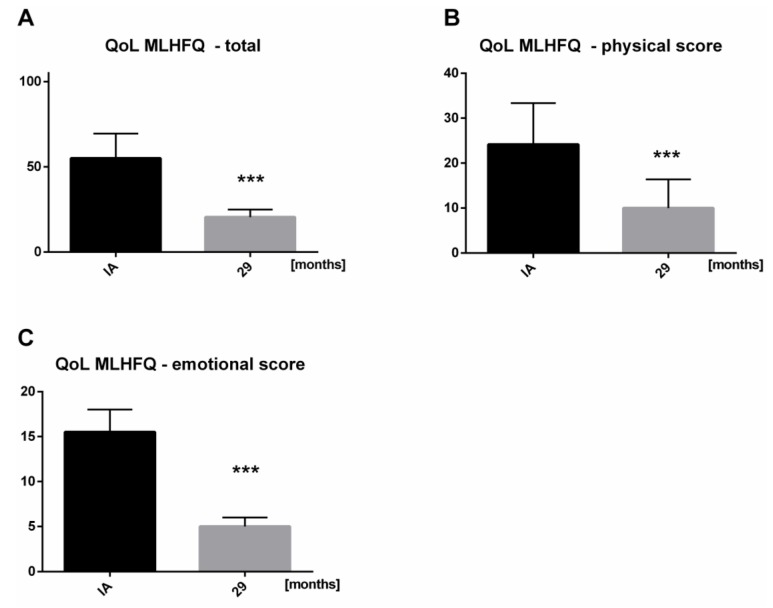
Quality of life (QoL) Minnesota Living with HF Questionnaire (MLHFQ) assessment results before immunoadsorption and at long-term follow-up. Left bars represent value before immunoadsorption and right bars at follow-up 3. (**A**) Total score (n = 22, *** *p* < 0.0001). (**B**) Subcategory of physical factors (*** *p* < 0.0001). (**C**) Subcategory of emotional factors (*** *p* < 0.0001). (Initial diagnosis (ID), immunoadsorption (IA)).

The MLHFQ median at baseline was 55.0 points and demonstrated a significant improvement to 21.0 points at long-term follow-up (*p* < 0.0001) (Figure 5A). In addition, the physical score showed a significant decrease from 27.0 points before immunoadsorption to 8.5 points (*p* < 0.0001) (Figure 5B). Furthermore, immunoadsorption improved the participants’ emotional condition from 15.5 points to 5.0 points (*p* < 0.0001) (Figure 5C). The response rate to QoL evaluation was 67% at long-term follow-up. The remaining 11 patients, who did not answer the QoL questionnaire included one patient only participated in the first follow-up visit; this patient demonstrated an increase of LVEF from 19% to 42%. Nine patients showed a relative increase in LVEF of 20%, demonstrating maintained responsiveness. The remaining patient who did not answer the QoL evaluation was an echocardiographic non-responder.

## 4. Discussion

Recent-onset cardiomyopathy in patients is often associated with a very poor prognosis, even in the context of optimal, guideline-recommended HF therapy, due to a lack of causal therapeutic options. Therefore, we evaluated the impact of early immunoadsorption and subsequent IVIG administration in addition to guideline-recommended OMT on cardiac function and symptoms in previously healthy patients diagnosed with recent-onset HF. All patients received optimal, guideline-recommended pharmacological HF treatment, which was maintained for a median time period of 5.2 months. During this period, NYHA functional class improved, whereas LVEF remained unchanged at a severely reduced level. Consecutively, all patients received a single cycle of immunoadsorption with IVIG administration. The patients experienced a further and continuous significant improvement of NYHA class throughout the 29-month follow-up period. Patients showed a significant improvement of LVEF and LVD. Furthermore, a significant decrease was observed in NT-proBNP levels during long-term follow-up. These hemodynamic benefits were paralleled with the significant clinical improvement of NYHA class and QoL evaluation. Patients reported a significant improvement of QoL overall during the long-term follow-up, as well as for the sub-categories of physical and psychological condition.

### 4.1. Benefits of Immunoadsorption and Subsequent IVIG in Patients with Limited HF Therapy

The beneficial effects of immunoadsorption in patients with idiopathic DCM have been demonstrated in several trials over the last two decades. However, pharmacological treatment with beta-blockers and mineralocorticoid receptor antagonists was not recommended in former HF guidelines [14], limiting the optimal HF therapy to ACE-inhibitors and ARB, diuretics and digitalis. Müller et al. demonstrated an improvement of LVEF from 22 to 38% after sole immunoadsorption without subsequent IVIG substitution in patients with idiopathic DCM in a 1-year follow-up. Similarly, additional IVIG administration following immunoadsorption in chronic HF led to a beneficial effect on LVEF [15,16]. Felix et al. performed a complex, invasive and time-consuming immunoadsorption protocol of four monthly cycles of immunoadsorption, each consisting of 2–3 sessions and followed by IVIG to improve NYHA class, as well as cardiac output in DCM patients. Importantly, in contrast to our cohort, patients in both studies did not receive a mineralocorticoid receptor antagonist therapy [17] and only partly a beta-adrenergic blocking therapy [14], which is recommended according to current HF guidelines [8,9,18]. In our trial, patients were treated with state-of-the-art pharmacological HF therapy: All patients received ARB/ACE-inhibitor therapy and beta-blocker therapy, and 97% of the study population received mineralocorticoid antagonist therapy. Moreover, former studies used different immunoadsorption protocols. Staudt et al. compared two different protocols of immunoadsorption treatment in a randomized study [19]: One group of patients was treated with four immunoadsorption courses at monthly intervals, the other group received one immunoadsorption course only, without repetition. Every immunoadsorption course was a cycle of five days. In a six-month follow-up, both groups showed a comparable effectiveness. Beware of these results: we performed a simplified immunoadsorption protocol, with a single cycle of immunoadsorption consisting of five daily sessions followed by a single IVIG administration to limit invasiveness. Furthermore, Staudt et al. evaluated IgG columns with high affinity to the IgG3 (Therasorb^®^) autoantibody subclass as more effective in dilated cardiomyopathy than a Protein A column based strategy [20,21]. The Protein A column we and others [13] used has a lower IgG3 affinity and there was no specific IgG3 subclass monitoring in our treatment approach.

### 4.2. Immunoadsorption and Subsequent IVIG Administration in Recent-Onset Cardiomyopathy vs. End-Stage Heart Failure

In previous trials [15,17,19], patients suffering from end-stage heart failure for several years were included after prolonged conservative pharmacological treatment, since all other therapeutic options had been exhausted. Müller et al. still observed an improvement of left ventricular function after immunoadsorption in 17 patients with idiopathic DCM three years after symptom-onset, compared to a control group on OMT during 12-month follow-up. After immunoadsorption therapy, patients showed a benefit in LVEF, left ventricular diameters, NYHA class and autoantibodies against β1-adrenoreceptors. [17]. Felix et al. demonstrated clinical and functional improvement after four years disease course [15]; likewise, Staudt et al. found improvement of LVEF in DCM patients even five years after symptom-onset [19]. In our trial, immunoadsorption and IVIG treatment was performed as early as 5.2 months on OMT after initial diagnosis of DCM and led to a significant improvement in symptoms and cardiac function. Findings in former clinical trials indicate first beneficial effects of OMT after one to three months [22,23] and a favorable short-term prognosis of recent-onset idiopathic DCM or myocarditis without therapeutic interventions up to six months [24]. Hence, in our study population no further benefit was expected by a prolonged conservative HF treatment.

### 4.3. Predictors for Responsiveness to Immunoadsorption

The identification and determination of predictors for responsiveness to immunoadsorption is of high clinical relevance to warrant selective treatment of DCM patients with this invasive, time- and resource-consuming therapeutic option. In several studies clinical parameters, autoantibodies and myocardial gene expression were evaluated to determine predictors of response to immunoadsorption [13,25,26]. Response to treatment is generally considered as improvement of NYHA class and cardiac function during the first months after immunoadsorption [26]. Response rates to immunoadsorption therapy show a wide inter-individual variability [25] and range between 48% [13] and 79.6% [26]. Applying the response criteria by Ohlow et al., including the improvement of ≥2 parameters of NYHA, LVEF, LVDd, MLHFQ QoL score, NT-proBNP levels and tolerated workload during stress testing in our cohort with recent-onset cardiomyopathy, the response rate was 100%. Focusing on maintained echocardiographic LV contractility improvement (≥20% of the pre-immunoadsorption value) as the main criterion for responsiveness to treatment, 90% of patients demonstrated responsiveness to IA and IVIG therapy. Ameling et al. determined short duration of DCM to be associated with responsiveness to immunoadsorption therapy. In their analysis, the mean disease duration of responders was 16 months, compared to 52 months in non-responders [25]. Analogous outcomes regarding the disease duration were presented in trials by Dandel et al. [26] and Ohlow et al. [13]. In our trial, median time period from symptom-onset to immunoadsorption therapy was as short as 5.2 months, suggesting the short disease course might have contributed to the remarkable response rate.

Prior studies reported that the presence of anti-cardiac autoantibodies and a low affinity Fcγ receptor genotype were predictors for a higher response rate [21,27]. We focused on clinical parameters to select patients for immunoadsorption and IVIG treatment. Besides the recent-onset of HF symptoms, we focused on patients’ recent medical history. The majority of previously healthy patients reported a history of infectious disease preceding the recent onset of HF symptoms by 1.4 months, thus suggesting a post-inflammatory cardiomyopathy. Importantly, normal white blood cell count and C-reactive protein levels excluded an acute infectious disease at initial diagnosis of HF. Until recently, studies investigating the effect of immunoadsorption in idiopathic DCM had excluded patients with suspected post-inflammatory cardiomyopathy [26]. Nevertheless, Ohlow et al. determined predictors of responsiveness to immunoadsorption in 91 patients with unspecific dilated cardiomyopathy including post-inflammatory cardiomyopathy [13]. In their analysis, Ohlow et al. identified myocardial inflammation as an independent predictor for response to immunoadsorption [13], further supporting our results.

### 4.4. Study Limitations

The main limitation of the study is the absence of an external control group to exclude the effect of OMT definitively. However, as a proxy, we employed each patient as an internal control using the phase of OMT from initial diagnosis to immunoadsorption. Only patients without further LVEF or NYHA class improvement by OMT were eligible for immunoadsorption therapy. Knowing the limitation of the absence of an external control group, we emphasized the precise description of the obtained medication and the respective dosage, changes of LVEF, NT-proBNP levels and NYHA class during the internal control phase on OMT for a median time of 5.2 months. Moreover, the study provides no information about presumed autoantibodies in the serum of the patients who underwent immunoadsorption therapy, so the patients were solely treated based on clinical symptoms. In addition, we cannot provide any genetic data concerning mutations in heart structure relevant proteins of the study population.

The strength of the study is the long-term cohort follow-up period of 29 months, with information about NYHA functional class, cardiac function, NT-proBNP levels and QoL after immunoadsorption treatment.

## 5. Conclusions

Administration of a single-cycle immunoadsorption of five days, in combination with subsequent IVIG substitution shortly after diagnosis of recent-onset HF, leads to a long-term improvement of symptoms, cardiac function, natriuretic peptide levels and QoL in addition to current pharmacological HF therapy. Selection criteria, based on the recent history of infectious disease linked to the recent-onset of HF symptoms, might help to identify potential responders by clinical criteria and improve long-term outcomes after immunoadsorption and subsequent IVIG administration.

## Figures and Tables

**Table 1:** Baseline characteristics at initial diagnosis of heart failure (HF).

Patients, n	35
Age, y	47.1 (35.1–53.8)
Sex, n	
Male	24
Female	11
Medical history	
History of preceding infectious disease	26 (74.3%)
Duration between infectious disease and HF, months	1.4 (0.6–2.9)
Lab values	
Troponin T, ng/L	22 (13–34)
NT-proBNP, ng/L	5720 (1850–7294)
White blood cell count, 10^9^/L	8.4 (6.9–9.9)
C-reactive protein, mg/L	4.8 (3.0–14.8)
Coronary artery disease (CAD)	
no CAD	23 (65.7%)
1-vessel CAD	8 (22.9%)
2-vessel CAD	4 (11.4%)
3-vessel CAD	0
Endomyocardial biopsy, n	27
Virus positive	13 (48.1%)
Cardiac magnetic resonance imaging (MRI), n	18
Diagnostic criteria for myocardial inflammation	13 (72.2%)

Values are n, n (%), median (IQR). IQR = interquartile range, NT-proBNP = N-terminal pro brain natriuretic peptide.

**Table 2:** New York Heart Association (NYHA) functional class and echocardiographic parameters at time point of initial diagnosis and immunoadsorption.

	Initial Diagnosis	Immunoadsorption	*p*-Value
Patients, n	35	35	
NYHA classification	3.0 (2.5–3.3)	2.0 (1.3–2.8)	<0.001
NYHA classification, n (%)			
I	0 (0.0)	9 (25.7)	
II	5 (14.3)	13 (37.1)	
III	21 (60.0)	12 (34.3)	
IV	9 (25.7)	1 (2.9)	
LVEF, %	27.0 (24.3–30.0)	27.0 (23.0–31.0)	0.80
LVDd, mm	67.0 (63.0–70.0)	65.5 (61.0–68.5)	0.31
LVDs, mm	57.0 (49.0–60.0)	51.0 (51.0–51.0)	0.50

Values are n, n (%), median (IQR). IQR = interquartile range, LVDd = left ventricular end-diastolic diameter, LVDs = left ventricular end-systolic diameter.

**Table 3:** Pharmacological HF treatment at initiation of immunoadsorption and % of dose equivalent of each substance.

Patients, n	35
Heart rate (HR), bpm	70 (62.0–74.5)
ARB, n	9
% of dose equivalent	25.0 (25.0–50.0)
ACE-inhibitor, n	26
% of dose equivalent	50.0 (44.4–100.0)
Beta-adrenergic blocking agent, n	35
% of dose equivalent	50.0 (25.0–75.0)
Aldosterone-antagonist, n	34
% of dose equivalent	50.0 (50.0–50.0)
Loop diuretics, n	29
Digitalis, n	2
Cardiac resynchronization therapy, n	2

Values are n, median (IQR). IQR = interquartile range, ARB = angiotensin receptor blocker, ACE = angiotensin converting enzyme.
